# Reconstruction of a Soil Microbial Network Induced by Stress Temperature

**DOI:** 10.1128/spectrum.02748-22

**Published:** 2022-08-16

**Authors:** Dailin Yang, Hiromi Kato, Kazutaka Kawatsu, Yutaka Osada, Toyohiro Azuma, Yuji Nagata, Michio Kondoh

**Affiliations:** a Graduate School of Life Sciences, Tohoku University, Sendai, Japan; b Faculty of Science, University of Tokyo, Tokyo, Japan; University of Minnesota

**Keywords:** metabolic theory of ecology, stress gradient hypothesis, empirical dynamic modeling, interaction network, soil microbiota, stability

## Abstract

The microbial community is viewed as a network of diverse microorganisms connected by various interspecific interactions. While the stress gradient hypothesis (SGH) predicts that positive interactions are favored in more stressful environments, the prediction has been less explored in complex microbial communities due to the challenges of identifying interactions. Here, by applying a nonlinear time series analysis to the amplicon-based diversity time series data of the soil microbiota cultured under less stressful (30°C) or more stressful (37°C) temperature conditions, we show how the microbial network responds to temperature stress. While the genera that persisted only under the less stressful condition showed fewer positive effects, the genera that appeared only under the more stressful condition received more positive effects, in agreement with SGH. However, temperature difference also induced reconstruction of the community network, leading to an increased proportion of negative interactions at the whole-community level. The anti-SGH pattern can be explained by the stronger competition caused by increased metabolic rate and population densities.

**IMPORTANCE** By combining amplicon-based diversity survey with recently developed nonlinear analytical tools, we successfully determined the interaction networks of more than 150 natural soil microbial genera under less or more temperature stress and explored the applicability of the stress gradient hypothesis to soil microbiota, shedding new light on the well-known hypothesis.

## INTRODUCTION

Soil microbiota play essential roles in diverse ecosystem processes (1–5), such as nutrient cycle (6), carbon cycle (7, 8), pollutant degradation (9, 10), plant growth promotion (11, 12), and climate regulation (13). Moreover, the structure of the interaction network has been related to the function and dynamics of the microbial community (14–18), which agrees with community ecological theories (19). Besides, with the help of network science tools, the community's network structure has also been used to identify functionally essential species in microbial ecology (20–23).

The stress gradient hypothesis (SGH) (24) relates the community network structure to environmental stress. It predicts that interspecific interactions shift to more positive ones under more stress (14, 16). At least two mechanisms exist that contribute to this pattern. The first mechanism is related to the altered community composition (that is, node changes in the community network) induced by stress (25, 26). Here, suppose the species that become extinct due to stress had more negative or less positive interactions, or the established species had more positive or less negative interactions: such species shifts would result in the pattern predicted by the SGH. The second mechanism is related to altered interspecific interactions (link changes) (27–29). Here, differences in environmental factors lead to differences in species needs or capabilities (30) and cause qualitative changes (interaction switches) in interspecific interactions (31–33). If an environmental stress turns negative interactions into positive ones, again this leads to the pattern predicted by the SGH. Thus, the difference in interspecific interactions and community networks induced by environmental stress would be understood as a combined effect of the above-mentioned node and link changes.

However, detection and quantification of interspecific interactions and determination of community network structure are nontrivial challenges. For example, it has been reported that a minimal quantity of soil contains thousands of microorganisms (34–36). Furthermore, the types of interspecific interactions that connect microorganisms are diverse, including competition, feeding relationships, mutualism, and commensalism (37–39). Hence, their identification can be a challenging task. Although many attempts have been made to construct “co-occurrence networks,” their ecological interpretation is unclear as co-occurrence does not necessarily mean the presence of dynamical interactions (40–42). Thus, a better understanding of how microbial community networks respond to environmental difference would be built on a better methodology to detect and quantify interspecific interactions.

Nonlinear time series analysis is a powerful approach to identify and quantify interactions in a complex ecological community (41–43). State-space reconstruction based on time delay embedding, a common practice in nonlinear time series analysis, allows one to use a single time series as proxy variables to reconstruct the attractor of the whole system. This technique has several applications, such as causality testing, forecasting, and tracking interaction strength and system stability (41, 44). Given the availability of quantitative time series in high-throughput sequencing for microbial communities, nonlinear time series analysis can be a suitable approach for constructing credible soil microbial interaction networks (45).

Here, by combining a controlled experiment involving a soil microbial community, high-throughput sequencing technology, and nonlinear time series analysis tool, we compared interspecific interactions and community network structures of a microbial community at 30°C and 37°C. Subsequently, microbial networks with more than 150 genera were inferred from the data analysis. Their comparison enabled detection and quantification of differences in microbial community networks associated with different temperatures. Furthermore, investigations revealed a more stressful temperature induced an increase in the proportion of negative interaction and simplification of the network structure of soil microbial community. The observed change, which was opposite from the prediction by the SGH, was explained in terms of metabolic response to different temperatures.

## RESULTS

**FIG 1:**
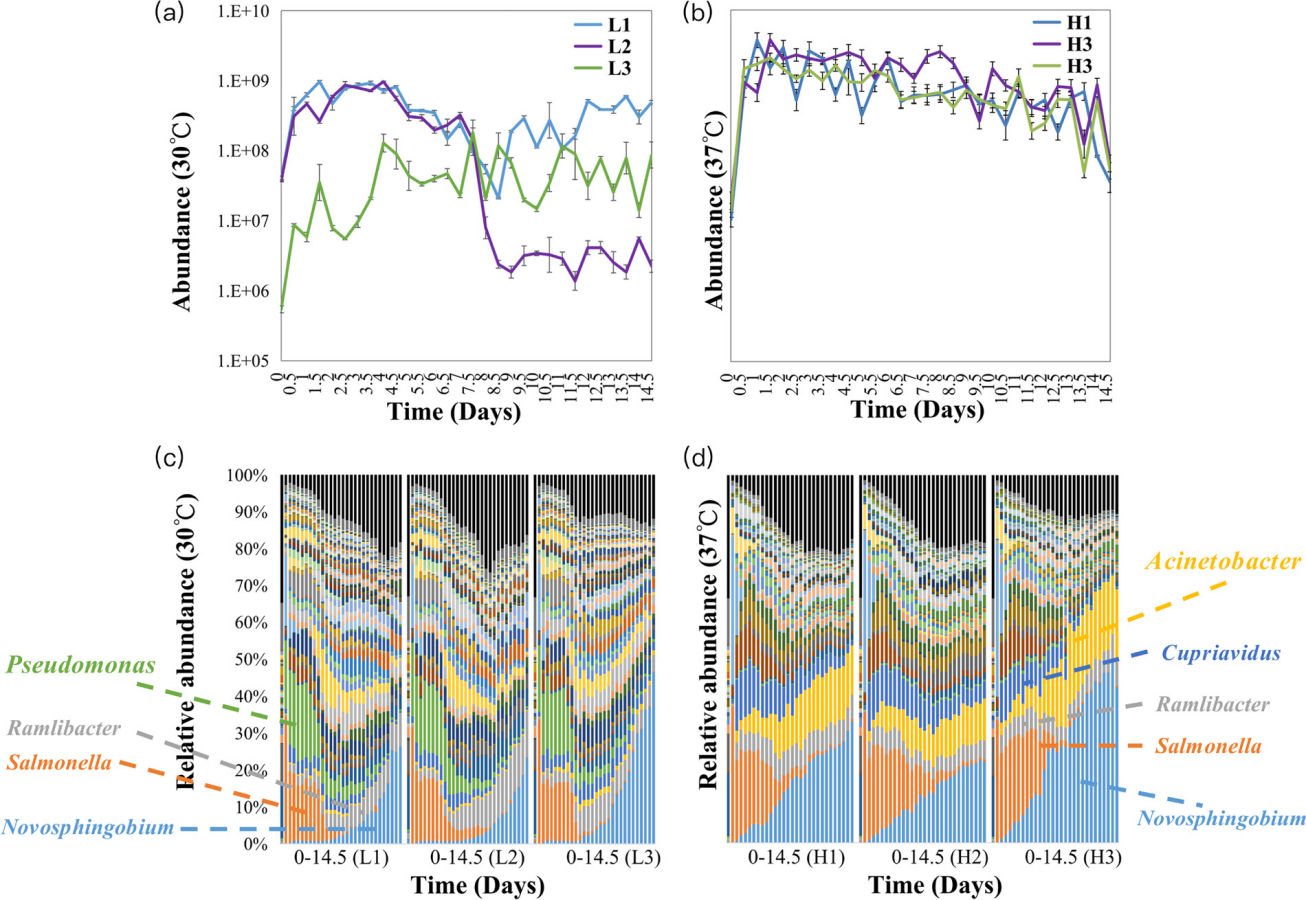
(a and b) Total abundance time series of soil microbial community at 30°C and 37°C. L1, L2, and L3 are mean samples incubated at 30°C. H1, H2, and H3 are mean samples incubated at 37°C. Three lines represent triplicate samples at the same temperature; the abundance base for logarithmic scales is base 10. (c and d) Relative abundance time series of community composition in soil microbial community at 30°C and 37°C. The different colored bars mean the proportion of the corresponding genus in total abundance. The complete figure is visible in the supplemental material.

We incubated a soil microbial community isolated from soil on liquid inorganic salt medium supplemented with soil extracts as a nutrient source and examined changes in total abundance and community compositions over time (14.5 days, with samples taken at 0.5-day intervals). The soil microbial community increased sharply in total abundance during the first 0.5 day. Then, it reached maximum abundance on days 1.5 to 4 and 1 to 1.5 at temperatures of 30°C (less stressful temperature) and 37°C (more stressful temperature), respectively (Fig. 1a and b). Results also showed that the incubation temperature affected the maximum total abundance and growth rate of the soil microbial community. As observed, the maximum total abundance increased from 9.6 × 10^8^ to 3.7 × 10^9^ copies/mL at the more stressful temperature and increased faster than the less stressful temperature in the first 0.5 day (Fig. 1a and b).

The temporal changes in compositions of genera were similar between the three replicates for the same temperature. As observed, Salmonella, *Ramlibacter*, and *Novosphingobium* were dominant genera in the soil microbiota at 30°C and 37°C; in addition, Pseudomonas was also dominant at 30°C, whereas Acinetobacter and *Cupriavidus* were also dominant at 37°C. The proportion of each component in the sample changed gradually over time (Fig. 1c and d).

**FIG 2:**
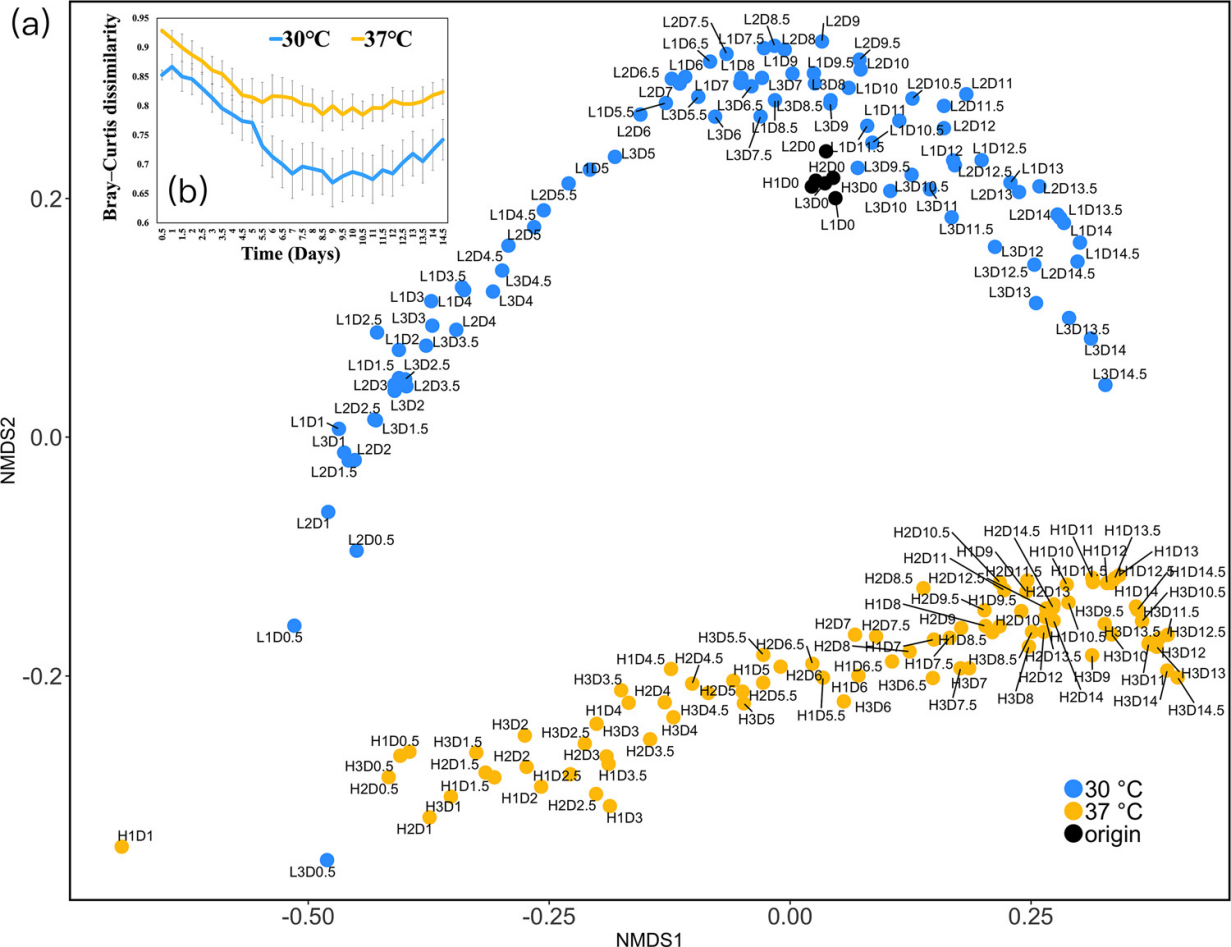
(a) Nonmetric multidimensional scaling based on genus-level amplicon data and Bray-Curtis dissimilarity (2 dimensions, stress is 0.108). The points represent samples taken every 0.5 day, and “D” in the tags represents the time of sampling. The closer the planar distances of the points, the more similar the community composition. (b) Bray-Curtis dissimilarity time series between original community and communities incubated at 30°C or 37°C, calculated based on genus-level data with no scaling.

Bray-Curtis dissimilarity from the original community (day 0) was higher for communities incubated at 37°C than at 30°C, confirming that the higher temperature caused a larger shift in community composition and is more stressful to the soil microbial community (Fig. 2a and b).

**FIG 3:**
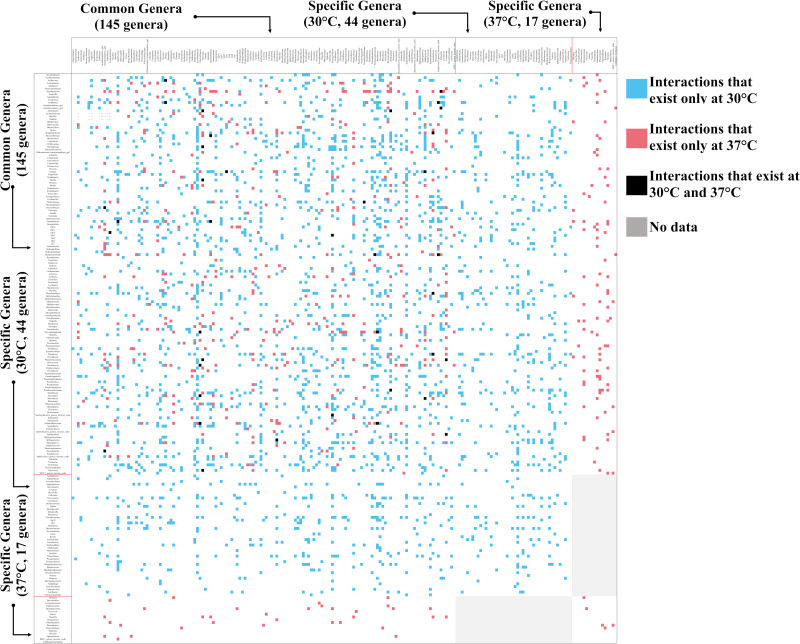
Interaction networks at 30°C and 37°C. Blue points represent interactions that exist only at 30°C, red points represent interactions that exist only at 37°C, and black points represent interactions that exist at both 30°C and 37°C. Gray shading indicates no data. The genera listed at the top of the figure represent genera that received interactions, whereas genera listed at the left of the figure represent genera with the given interactions. The complete figure is visible in the supplemental material.

Interactions were inferred by applying nonlinear time series analysis (UIC, S-map) to the genera present at more than 47 time points. The constructed network structure was less complex for the 37°C condition: the number of genera (in addition to the 145 common genera, 44 30°C-specific genera and 17 37°C-specific genera were observed) was decreased by 14.3%, and connectance was lowered by 67.1% (0.068 at 30°C and 0.022 at 37°C) (Fig. 3).

The proportion of positive interactions was higher at 30°C (2,241/2,409 [93.03%]) than at 37°C (394/581 [67.81%]) (Table 1). In addition, the proportion of pairs that have at least one positive interaction and do not have a negative interaction ([+, 0] or [+, +]) was higher at 30°C (2,120/2,288 [92.66%]) than at 37°C (391/576 [67.88%]) (Table 1). Similarly, the proportion of pairs that have at least one negative interaction and do not have positive interaction ([−, 0] or [−, −]) was lower at 30°C (161/2,288 [7.04%]) than at 37°C (184/576 [31.94%]) (Table 1). Similar patterns were also observed in the common subnetworks of genera. Specifically, the proportion of positive interactions was higher at 30°C (1,518/1,625 [93.42%]) than at 37°C (292/450 [64.89%]) (Table 1). In addition, the proportion of pairs that have at least one positive interaction and do not have a negative interaction ([+, 0] or [+, +]) was higher at 30°C (1,423/1,530 [93.00%]) than at 37°C (291/447 [65.10%]) (Table 1). The proportion of pairs that have at least one negative interaction and do not have a positive interaction ([−, 0] or [−, −]) was lower at 30°C (100/1,530 [6.54%]) than at 37°C (156/447 [34.90%]) (Table 1).

**TABLE 1:** Distribution of interaction relationships between pairs of genera of the complete network and common subnetwork and distribution of the proportion of interactions between the complete network and common subnetwork at 30°C and 37°C^*a*^

Distribution type	Interaction sign(s)	Proportion (%) at:		Possible state
		30°C	37°C	
Interaction between relationships of pairs of genera				
Complete network	(+, +)	4.98	0.35	Mutualism
	(+, 0)	87.67	67.53	Commensalism
	(+, −)	0.31	0.17	Antagonism
	(−, 0)	7.04	31.60	Amenalism
	(−, −)	0.00	0.35	Competition
Common subnetwork	(+, +)	5.75	0.22	Mutualism
	(+, 0)	87.25	64.88	Commensalism
	(+, −)	0.46	0.00	Antagonism
	(−, 0)	6.54	34.45	Amenalism
	(−, −)	0.00	0.45	Competition
Interactions between complete network and common subnetwork				
Complete network	+	93.03	67.81	
	−	6.97	32.19	
Common subnetwork	+	93.42	64.89	
	−	6.58	35.11	

a The positive (+) and negative (−) interactions between the pairs of genera and complete network and common subnetwork are shown, along with the percentages indicating the proportion of pairs of that state out of all pairs with interactions. “Possible states” represent the ecological relationship of the pair.

**FIG 4:**
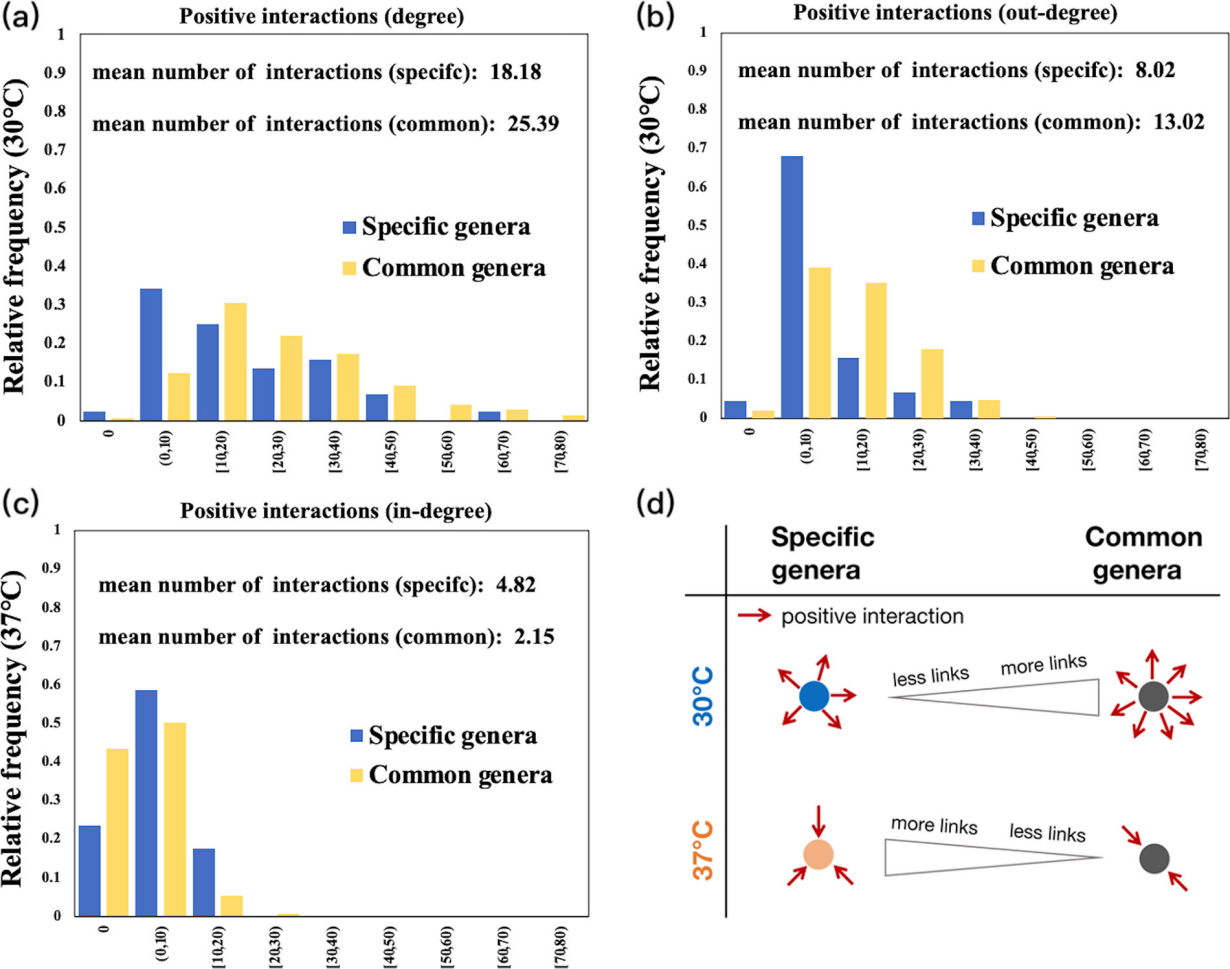
(a, b, and c) Distribution of the degrees, in-degrees, and out-degrees of positive and negative interactions of the specific and common genera in the complete network. The out-degree and in-degree, respectively, represent that the node gives or receives interactions, whereas the degree includes both receiving and giving interactions. Only statistically significant results are shown here. (d) Diagram of statistically significant characteristics of specific and common genera at 30°C and 37°C.

At 30°C, the degree and out-degree of positive interactions were significantly lower for the specific genera than for the common genera (at *P* < 0.01, the mean degrees of positive interactions for the common and specific genera were 25.39 and 18.18, respectively; at *P* < 0.001, the mean out-degrees of positive interactions for the common and specific genera were 13.02 and 8.02, respectively) (Fig. 4a and b), and no significant difference was observed in the in-degree of positive interactions (*P* = 0.21). There were no significant differences in degree, in-degree, or out-degree of negative interactions between the common and specific genera (*P* = 0. 34, *P* = 0.33, and *P* = 0. 44, respectivley). At 37°C, the in-degrees of positive interactions were significantly higher for the specific genera than for the common genera (at *P* < 0.05, the mean in-degrees of the positive interactions for the common and specific genera were 2.15 and 4.82, respectively) (Fig. 4c), and no significant differences were observed in the degree or out-degree (*P* = 0.39 and *P* = 0.08, respectively); there were also no significant differences in degrees, in-degrees, or out-degrees of negative interactions between the common and specific genera (*P* = 0.37, *P* = 0.94, and *P* = 0.42, respectively). The significant results are summarized in Fig. 4d.

No significant structural similarities were identified between microbial networks under different temperatures. Specifically, no significant correlations were observed for the common genera at 30°C and 37°C in the degrees, in-degrees, or out-degrees (Spearman’s ρ = 0.261 and *P* < 0.01, ρ = 0.120 and *P* = 0.149, and ρ = 0.141 and *P* = 0.091 for degrees, in-degrees, and out-degrees, respectively). Similarly, no significant product-moment correlation was observed between subnetworks of common genera at different temperatures (*r* = 0.0037, *P* = 0.275).

## DISCUSSION

In the present study, the soil microbial community was incubated at 30°C and 37°C. There are several lines of evidence that 37°C was more stressful condition than 30°C. First, the community composition was closer to the original obtained in the field at 30°C than at 37°C (Fig. 2a and b), suggesting that the higher temperature acted as a stronger disturbance and thus can be interpreted as a stronger stress to the microbiota. Second, according to the Japan Meteorological Agency (https://www.data.jma.go.jp/obd/stats/etrn/view/daily_s1.php?prec_no=73&block_no=47887&year=2011&month=5&day=19&view=p1), the temperatures of the field site where the microbial community was sampled (Matsuyama City, Ehime Prefecture, Japan) ranged from 14.7 to 26.2°C on the day of sample collection (19 May 2011). In addition, the annual maximum temperature at the site was 35.9°C, which is lower than the high-temperature experimental condition (37°C), again suggesting that 37°C was likely to be stressful for the community we used. Based on the above evidence, 37°C should be a more stressful incubation temperature than 30°C for the soil microbial community.

The SGH predicts that more stressful environment favors positive interactions (24, 46). However, contrary to the hypothesis, the present study revealed that interspecific interactions of the incubated soil microbial community became more negative under higher temperature stress. More specifically, the proportion of mutualism and commensalism pairs decreased at 37°C, whereas amenalism and competition pairs increased (Table 1). Similarly, the proportion of positive interactions in the complete interaction network was lower at 37°C (Table 1).

In theory, SGH could arises from two mechanisms, including stress-induced extinctions (or establishments) of genera that have more negative or less positive (more positive or less negative) interactions (mechanism I) and stress-induced interaction switches that decrease the negative interactions or increase the positive ones (mechanism II). This study showed that the two mechanisms worked complexly to generate a pattern contrary to the SGH in the microbial community. Mechanism I operated in agreement with SGH: less stressful conditions enabled otherwise extinct genera with less outgoing positive interactions (Fig. 4a, b, and d), and more stressful situations enabled the establishment of genera that received more positive interactions (Fig. 4c and d). However, this result did not lead to more dominance of positive interactions under a more stressful temperature, as mechanism II intercepts to prevent the SGH from holding in this system. The genera in the common subnetwork, which had more positive interactions under less stressful conditions, shifted to more negative (or less positive) ones under the more stressful temperature (Table 1). Hence, mechanisms I and II combined increased negative interactions in the more stressful environment in the entire network, in opposition to the prediction of SGH.

The more stressful temperature simplified the community network structure (Fig. 3). As observed, the numbers of genera (nodes) and connectance showed 14.3% (189 to 162) and 67.1% (0.068 to 0.022) decreases, respectively, under the more stressful temperature. The simplification might be explained by the increased proportion of negative interactions. Increased competitive pairs can lower richness of genera by competitively excluding inferior genera. Moreover, ecological theory predicts that the community network complexity's effect on community stability varies with interaction types present in the community (19, 47). This theory implies that increased negative interactions under a more stressful temperature could change the complexity-stability relationship and induce changes in the community network's complexity. The simplification of the community network observed in the present study might be relevant to the theory.

The increased proportion of negative interactions and competitively interacting pairs under the more stressful temperature reflects the metabolic response of microorganisms (48). According to the metabolic theory of ecology (MTE), the metabolism and growth rate of individual species are faster in a high-temperature environment and more energy and materials are needed for the persistence of populations. Therefore, the supply of materials and energy cannot meet the high growth rate, the interspecific interactions become more competitive, and species are more proposed to become extinct (48–53). Hence, the higher maximum total abundance, faster growth, and higher proportion of negative interactions (Fig. 1a and b; Table 1) at 37°C than 30°C support the increased metabolic rate caused by higher temperature, consistent with the theory and earlier supporting evidence (54, 55).

Different temperatures induce complete difference in a network's structure, suggested by the absence of correlations between the degrees, in-degrees, or out-degrees of common genera in the complete interaction network. Moreover, the interaction network structures of the subnetwork of common genera was different at different temperatures. This finding proposes that network structure-based characterization of individual species is strongly condition dependent. It has been usual practice to specify keystone species or functionally important species from network topology (20, 56–58). However, such a practice can be misleading, given that the microbial interaction networks vary considerably from global to local levels under different environmental conditions (temperature). This study, however, demonstrates that temperature is a fundamental factor affecting environmental change that shapes interspecific interactions and soil microbiota network structures. Nevertheless, how those network structural changes affect the population at community-level dynamics, as predicted in community ecological theory, is essential for future studies.

The present result that higher stress favors negative interactions was, however, inconsistent with some previous studies looking at much-less-complex communities (14, 16, 59). Hesse et al. also showed that toxic copper promoted positive interactions in a 10-species compost microbial community (14), Piccardi et al. showed that toxicity drives facilitation between 4 bacterial species (16), and Yu et al. showed that nitrite, which acts as a stressor for the microbial community, enabled a 13-species microbial community of municipal wastewater to have fewer negative interactions (59). These studies are all consistent with the predictions of SGH, unlike the present study looking at the temperature stresses. The difference might be explained by the kind of environmental stress. If the increase in negative interaction under the high-temperature condition was due to the increased metabolism, the same pattern would not be observed under another kind of stress that has no such effects.

### Conclusion.

We showed that increased temperature stress induces a complex response of a microbial community network. The results are partially consistent with SGH (i.e., genera that became extinct under a more stressful temperature have fewer positive interactions in the outgoing direction, and new genera selected by more stressful temperature have more positive interactions in the incoming direction). However, overall network changes were contrary to the predictions of SGH, with the interaction network moving in a more negative direction under the more stressful temperature, mainly due to interaction switches between common genera.

## MATERIALS AND METHODS

### Microbiota.

The microbial community was obtained from a farm soil (soil type, brown forest soil) at the Ehime Research Institute of Agriculture, Forestry, and Fisheries, in Matsuyama, Japan (60). The soil was sampled at depths of 5 to 10 cm from the surface on 19 May 2011, and large particles were removed with a 2-mm-mesh sieve. The soil consisted of 75% sand, 12% silt, and 13% clay. Therefore, it was classified as sandy loam. Microbial cells were isolated from the soil sample as follows. First, the soil was suspended in phosphate-buffered saline (PBS) and homogenized using a blender. Then, large soil particles were allowed to settle by weak centrifugation (420 × *g*, 10 min, 10°C), and cells in the supernatant were transferred to a flask. Afterward, the precipitated soil particles were subjected to two more sets of cell isolation, after which a pooled cell suspension in the flask obtained from 1 kg of the soil was filtered (pore size, 7 μm) (Advantec, Tokyo, Japan) to remove small soil particles and centrifuged (8,000 × *g*, 20 min, 10°C) to obtain cell pellets. Finally, cell pellets were suspended in PBS and used as the inoculum of the microbial community.

### Growth conditions.

First, 2 mL of the extracted microbial community was inoculated into 200 mL of the liquid medium in a Sakaguchi flask and incubated at 30°C and 37°C with shaking in triplicate, respectively. While the three samples incubated at 30°C were named L1, L2, and L3, three other samples incubated at 37°C were named H1, H2, and H3, respectively. Soil extract was added to ensure maximum culturability (61, 62). The soil extract was prepared by autoclaving 400 g of the soil suspended in 1 L distilled water at 121°C for 30 min and then filtered through no. 2 filter paper (Advantec). The composition of the liquid medium was as follows: 1/10-strength W medium (63) [KH_2_PO_4_, 170 mg; Na_2_HPO_4_, 980 mg; (NH_4_)_2_SO_4_, 100 mg; MgSO_4_, 48.7 mg; FeSO_4_, 0.52 mg; MgO, 10.75 mg; CaCO_3_, 2.0 mg; ZnSO_4_, 0.81 mg; CuSO_4_, 0.16 mg; CoSO_4_, 0.15 mg; H_3_BO_3_, 0.06 mg/L] containing 10% of the soil extract as a carbon source.

### Sampling and DNA extraction.

The culture medium (1.8 mL) was sampled every 0.5 day and stored in 15% glycerol stock (−80°C) until 14.5 days starting from day 0. To standardize the extraction efficiency, we added an artificial DNA containing the enhanced green fluorescent protein (EGFP) gene as an internal standard (64) to the samples before extracting DNA using the DNeasy PowerSoil kit (Qiagen, Hilden, Germany). DNA was extracted according to the manufacturer's instructions.

### High-throughput sequencing of 16S rRNA amplicons.

To amplify the hypervariable V3-V4 region of the 16S rRNA gene, 2× Gflex PCR buffer (Mg^2+^, deoxynucleoside triphosphate [dNTP] plus), Tks Gflex DNA polymerase (1.25 U/μL), and primers 342F (10 μM) and 806R (10 μM) (65) appended to overhanging adaptor sequences for Nextera XT indices (Illumina) were used for PCR as follows. We amplified the target region using 29 cycles of denaturing at 98°C for 15 s, annealing at 55°C for 30 s, and extension at 68°C for 30 s. Subsequently, PCR products were purified using AmPure XP. Then, amplicon libraries were indexed with a Nextera XT index kit (Illumina), followed by sequencing with an Illumina Miseq platform, using a Miseq reagent kit v3 (300 cycles, paired end).

### Taxonomic assignment of the sequence.

A total of 26,877,620 pairs of raw reads (average 140,000 pairs/sample) generated using the Miseq sequencer were quality controlled, clustered, and assigned using the UPARSE pipeline (66) and RDP classifier (67) with the following criteria. First, the raw reads were trimmed with a quality score of ≤30 and merged (with minimum value of mismatches and overhang of 10 and 30, respectively). Then, both primer regions of the merged reads were removed with Tagcleaner (68). Next, the merged reads containing *N*, including reads derived with the PhiX control, and reads with an expected error of more than 1 base/read were removed. The resultant high-quality reads were subjected to dereplication to obtain unique reads. Unique reads without singletons were clustered into 97% operational taxonomic units (OTUs), and chimeras were checked by using the default setting of cluster_otus command in UPARSE. The obtained OTUs were assigned to each taxonomic level based on the RDP classifier (67). The taxonomic composition of each sample was determined based on the mapping of the merged reads onto the OTU data set. We preferred to use the smallest possible taxonomic unit to construct the network to make the interaction network's taxonomic resolution as fine as possible. However, since the smallest taxonomic unit (OTU) had a small read count and was susceptible to observation noise, we used the genus level to construct the interaction network.

### Real-time PCR.

We determined the total abundance of microbial community using real-time PCR with the CFX Connect real-time system (Bio-Rad). DNA extraction efficiency was determined by adding a known concentration of artificial DNA containing the EGFP gene as an internal standard (64): that is, we centrifuged 1 mL of the culture and discarded 750 μL of the supernatant, added 1 × 10^7^ copies of artificial DNA containing the EGFP gene to the precipitate, and performed DNA extraction. Then, we measured the residual amount of artificial DNA containing the EGFP gene after DNA extraction using real-time PCR to estimate the DNA extraction rate, using the DNA extraction rates to revise the abundance of microbial community. Luna Universal quantitative PCR (qPCR) master mix and primers 1070R (5′-AAGTCGATGCCCTTCAGCT-3′ [10 pM]) and 960F (5′-CCAGGAGCGCACCATCTT-3′ [10 pM]) were used to amplify the artificial DNA-containing EGFP gene. Also, primers 342F (5′-CTACGGGGGGCAGCAG-3′ [10 pM]) and 806R (5′-GGACTACCGGGGTATCT-3′ [10 pM]) were used to amplify the V3-V4 region of the 16S rRNA gene. The thermal cycle of the amplification was 39 cycles of denaturation at 95°C for 5 s, annealing at 55°C for 10 s, and extension at 68°C for 10 s; the thermal cycle settings of the artificial DNA containing the EGFP gene and 16S rRNA gene are the same.

### Determination of the stress condition.

To determine the magnitude of stress on soil microbial communities at 30°C and 37°C, we performed dissimilarity analyses of community composition at the genus level using Bray-Curtis dissimilarity (69) and nonmetric multidimensional scaling (70). Bray-Curtis dissimilarity was calculated using the vegan package (version 2.5-7), and nonmetric multidimensional scaling was performed using the “mass” package (version 7.3-54) for R (version 4.1.0). We also used temperature data to determine the magnitude of stress: as recorded in meteorological data from the Japan Meteorological Agency (https://www.data.jma.go.jp/obd/stats/etrn/view/daily_s1.php?prec_no=73&block_no=47887&year=2011&month=5&day=19&view=p1), the temperature in Matsuyama City, Ehime Prefecture, on the day of sample collection (19 May 2011) ranged from 14.7 to 26.2°C, and the annual maximum temperature was 35.9°C in 2011.

### Construction of the interaction network.

The absolute abundance of the genus (*i*) at a time point (*t*), *N_i_* (*t*), was calculated as *N_T_* (*t*) *P_i_* (*t*), where *N_T_* (*t*) was the total abundance of soil microbial community measured by real-time PCR and *P_i_* (*t*) was the proportion of genus *i*’s abundance measured by the 16S rRNA gene's amplicon sequencing. Samples of each temperature in triplicate were analyzed for 90 time points (30 time points per sample; *n* = 3). In order to avoid dynamic disturbances of the initial sampling temperature and ensure the attractor's accuracy, we removed the data from day 0 (1 time point per sample; *n* = 3) and the genera that were absent for more than 40 time points of the remaining 87 time points.

We used a nonlinear causality test, UIC, to identify the interaction between various soil microbiota genera. UIC is based on the state-space reconstruction founded on the Takens embedding theorem, and it was developed based on CCM (42, 71). UIC detects causality from the time series by examining the information flow (transfer entropy) (72) between focal variables (71). More specifically, species X is judged to have causality to species Y at time lag Δ*t* if the inclusion of species Y's data at time *t* (Y*_t_*) during the state-space reconstruction improves the forecast skill of species X at time *t* − Δ*t* (X*_t_*_− Δ_*_t_*). The three time series obtained from replicates at each temperature were used in this study (73). We assumed no interactions between the genera when the existence of interaction cannot be statistically determined. Interaction inference was conducted using the wrapper function “uic.optimal” of the rUIC package (version 0.1.5), based on a multivariate simplex projection to automatically determine the embedding substratum and infer interactions. To avoid indirect interactions due to a long time lag as much as possible, we fixed the time lag to −1 (i.e., 0.5 day). Then, results were verified as significant using bootstrap and surrogate data (74). Furthermore, a regularized S-map (75) was used to determine the strength and sign of individual interactions. For this investigation, we used the mean of the S-map coefficients at each time point to represent the strength of the interactions between the two genera.

### Interaction network analysis.

The network complexity was measured using the number of genera and connectance (the probability of two randomly chosen genera interacting). Based on the constructed network structure, all genera that appeared in the network were classified into three subgroups: 30°C-specific genera, which existed only at 30°C; 37°C-specific genera, which existed only at 37°C; and common genera, which existed at both temperatures. Subsequently, individual nodes were characterized with their degrees (the sum of all links connected to a node), in-degrees (the sum of incoming links connected to a node), and out-degrees (the sum of outgoing links connected to a node) (76, 77).

To test the constancy in the complete network's structure, Spearman's ρ (78) was evaluated for the degrees, in-degrees, and out-degrees of individual common genera in each complete network at 30°C and 37°C. The product-moment correlation (79) was evaluated between the subnetworks of common genera at 30°C and 37°C. (Interaction strength sign information is not included.) The product-moment correlation between matrices was calculated using the package sna (version 2.6) (80). Additionally, we explored sign changes of the complete network, subnetwork of common genera, and differences in interaction numbers between specific and common genera at 30°C or 37°C. The Wilcoxon rank sum test was used to test the statistical significance of the difference, which was implemented with self-contained package stats for R (version 4.1.0).

### Data availability.

The 16S rRNA gene sequence data of soil microbiota cultured at 30°C and 37°C have been deposited in DDBJ DRA under the project accession no. PRJDB11844 (81). The analysis data have been deposited in the Dryad database (82).
